# Coupling of a Quinoline Derivative and Rare-Earth-Doped TiO_2_ Thin Films: Prospective Application to Hybrid Light-Emitting Devices

**DOI:** 10.1021/acsomega.6c02539

**Published:** 2026-06-20

**Authors:** Natália Carli de Oliveira, Xavier Mateos, Vitor Fernandes Moreno, Luiz Carlos da Silva Filho, Luis Vicente de Andrade Scalvi

**Affiliations:** † São Paulo State University UNESP, School of Sciences, Chemistry Department and POSMAT, Avenida Engenheiro Luiz Edmundo Carrijo Coube 14-01, 17033-360 Bauru, Brazil; ‡ 16777 University Rovira I Virgili, Physical and Inorganic Chemistry Department, Tarragona 43007, Spain; § São Paulo State University UNESP, School of Sciences. Chemistry Department, Avenida Engenheiro Luiz Edmundo Carrijo Coube 14-01, 17033-360 Bauru, Brazil; ∥ São Paulo State University UNESP, School of Sciences, Physics and Meteorology Department, Avenida Engenheiro Luiz Edmundo Carrijo Coube 14-01, 17033-360 Bauru, Brazil

## Abstract

Hybrid organic–inorganic heterostructures offer a promising design toward low-cost light-emitting devices by combining the high luminescence efficiency of organic semiconductors with the stability and charge transport capability of metal oxides. In this work, TiO_2_ thin films doped with trivalent lanthanide ions (Er^3+^ and Yb^3+^, 2 and 4 at % concentrated) were prepared by the sol–gel dip-coating method and coupled to a quinoline-based donor–π–acceptor small molecule, 4-(6-(*diethylamino*)-4-*phenylquinolin*-2-*yl*) *benzoic acid*. X-ray diffraction confirmed preservation of the anatase phase after doping, while optical absorption revealed defect-related features and characteristic intra4f transitions of the lanthanides. Fourier-transform infrared spectroscopy demonstrated deprotonation of the carboxylic group and the formation of interfacial coordination bonds, with Er^3+^-doped TiO_2_ exhibiting a well-defined bridging bidentate configuration. Electroluminescence measurements showed diode-like behavior for all doped systems. The Er^3+^-doped TiO_2_ heterostructure exhibited a lower turn-on voltage (≈5 V), whereas the Yb^3+^-doped TiO_2_ system showed enhanced emission at a higher applied bias. A blue shift of the photoluminescence peak from 500 to 483 nm upon heterostructure formation indicates modification of the electronic environment of the excited state due to interfacial coupling. Photoinduced electrical measurements in the quinoline derivative (QD) layer show a slow recombination process consistent with local lattice relaxation and Variable Range Hopping (VRH) mechanism, with a characteristic recombination time of 59.2 s. These results demonstrate that rare-earth doping modulates interfacial coordination strength and charge injection dynamics, directly impacting the electroluminescent performance of TiO_2_/QD heterostructures.

## Graphical abstract

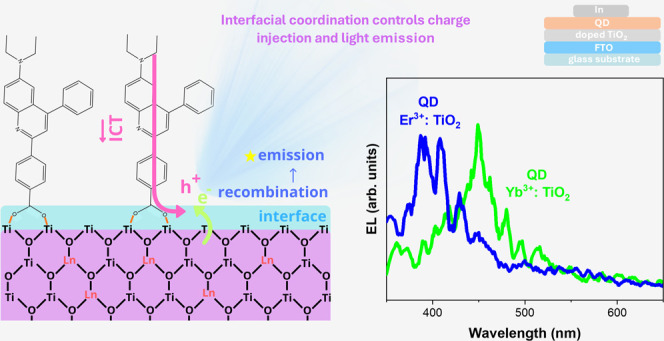

## 1 Introduction

Hybrid light-emitting diodes (LEDs) that combine organic and inorganic semiconductors have attracted considerable interest due to the possibility of merging complementary properties of both sorts of materials. Organic semiconductors may present high absorption coefficient, effective fluorescence,^1^ high luminous efficiency,^2^ and, in some systems, efficient charge transport.^3^ Inorganic semiconductors, on the other hand, stand out for high concentration of charge carriers, high electronic mobility, and conductivity, associated with physical and chemical stability.^4^ Hybrid LEDs represent a promising alternative to traditional inorganic devices, including liquid crystal displays,^5^ as they combine the high efficiency and broad color spectra of the organic emitting layer with low-voltage operation, in addition to the possibility of large-scale, lower-cost manufacturing using simple techniques such as the chemical routes.

Despite the significant progress in hybrid and organic-based optoelectronic devices, the integration of organic light-emitting layers with inorganic charge transport layers remains a major challenge. In particular, the electron transport layers can strongly influence charge injection, carrier balance, and interfacial recombination processes. Doping strategies have been widely employed to tailor the electrical properties of metal oxide transport layers.^6–8^ However, their impact on electroluminescence behavior and interfacial roles is still not fully understood. In many cases, modifications that enhance charge transport may simultaneously introduce localized states or accelerate degradation pathways, highlighting the need for a deeper understanding of how electronic transport layer (ETL) doping affects both optical response and device reliability.

Among the various metal oxides employed as ETL, titanium dioxide (TiO_2_) stands out due to its chemical stability,^9^ fair electronic mobility and efficient charge transport mainly in the anatase phase.^10^ Although rutile is the most thermodynamically stable phase and can be obtained at higher temperatures, it starts from the nucleation of anatase crystals.^11^ The anatase–rutile transition takes place at about 700 °C, where the Gibbs free energy of the anatase crystal becomes larger than that of rutile.^11,12^ Below this temperature of thermal annealing, such as the present case, anatase predominates. When doped with lanthanides (rare-earth) trivalent ions, such as Er^3+^ and Yb^3+^, it has been reported to promote the stability of the anatase phase,^13^ in addition to introducing intermediate energy levels within the bandgap, which can act as radiative recombination centers.^14,15^ Moreover, this doping may facilitate charge transfer at the interface with organic films, acting as an active layer for electron transport.

On the other hand, small organic molecules based on quinoline derivatives (QD) have attracted attention in optoelectronic applications due to their tunable electronic structure and light-emitting properties. The QD structure consists of a benzene ring fused to a pyridine ring, resulting in a π conjugated system with delocalized electrons, a characteristic that confers relevant optical properties. These compounds exhibit strong absorption in the ultraviolet and visible ranges^16^ and intense luminescence in the blue range.^17^ Besides, QD exhibits a characteristic Stokes shift, related to the energy difference between absorption and emission bands, which reflects the ability of the excited state to relax to a lower energy level before emitting photons.^18^ The QD used here is the *4-(6-(diethylamine)-4-phenylquinolin-2-yl)­benzoic acid*, where ligands such as diethylamine and phenol act as electron-donating. However, it is interesting to notice that the presence of benzoic acid, which exhibits 2 oxygen atoms, introduces electron-withdrawing characters for some parts of the molecule. This sort of behavior is called donor–π–acceptor (D–π–A) and may lead to interesting behavior related to optoelectronic devices. Although the main characteristic explored in the present paper is the acceptor nature, this double nature can be further investigated, since it attaches applicability potential to the QD deposited films. Isci and Ozturk^19^ proposed a D–π–A type compound, DMB-TT-TPA, containing triphenylamine as donor and dimesitylboron as acceptor linked through a thieno [3,2-*b*] thiophene as a π-conjugated linker. A rather large Stokes shift (>100 nm) was observed, being explained through a fast relaxation from the excited state to the ground state, as a result of intramolecular energy transfer between TPA and boron groups. Large Stokes shift has also been found by Ledwon et al.^20^ for similar structures, who proposed this type of dipolar chromophores molecule consisting of a D–π–A characteristic for integration in optoelectronic and electronic devices. Generally, the π-conjugated molecules provide substantial delocalization of π-electrons over the molecules,^21^ essential to fast carrier transport. The main conduction mechanism in these materials is variable range hopping (VRH).^22^ However, depending on the temperature and substituent groups, mechanisms such as small polaron tunneling (SPT), quantum mechanical tunneling (QMT), and correlated barrier hopping (CBH) can be observed.^23^ In this way, QD plays both roles as the hole transport layer (HTL) and light-emitting film as well. This multifunctional performance simplifies the device structure while enabling efficient coupling between charge transport and light emission. Understanding the intrinsic optical and electrical properties of the quinoline layer is therefore essential to explain its contribution to exciton formation, carrier dynamics, and overall device operation.

In this work, the obtainment of TiO_2_ doped with trivalent lanthanide ions (Er^3+^ or Yb^3+^) through a simple sol–gel dip-coating process is reported, along with film formation of QDs by the spin coating deposition technique. The coupling of this sort of material as heterostructures is also investigated, revealing a probable influence of lanthanide doping on the electro-induced light emission and performance of the resulting hybrid LEDs.

## 2 Experimental Section

### 2.1 Preparation of Solutions of Undoped and Doped TiO_2_

The TiO_2_ sol was obtained by a modified sol–gel route. Initially, 15 mL of isopropyl alcohol and 0.70 mL of 65% nitric acid (Synth) were added to 48 mL of distilled water under magnetic stirring. Then, 4 mL of titanium isopropoxide (IV) (Sigma-Aldrich) was slowly dropped into the mixture, which was kept stationary for 30 min. At this stage, the rapid formation of white precipitates was observed due to the hydrolysis of the alkoxide precursor.

For the preparation of doped TiO_2_ sols, erbium oxide (Er_2_O_3_, 99.9%, Sigma-Aldrich) and ytterbium oxide (Yb_2_O_3_, 99.9%, Sigma-Aldrich) were used as the dopant sources. The rare-earth concentrations were adjusted to achieve 0.5, 1.0, 2.0, 3.0, and 4.0 at. % relative to the incorporated titanium proportion. Each oxide was first dissolved in nitric acid (65%, Synth) under stirring with a magnetic bar, until a transparent solution was obtained. The acidic medium promotes the conversion of the oxides into nitrate forms, releasing free Er^3+^ or Yb^3+^ ions in solution, which facilitate their homogeneous incorporation into the TiO_2_ sol lattice.

The resulting rare earth nitrate solution was then added to the TiO_2_ sol. The mixture was stirred for 30 min until a bluish, translucent, and homogeneous solution was formed. Subsequently, 1 mL of Triton X-100 (Sigma-Aldrich) was added as a surfactant to enhance the colloidal stability and promote better adhesion and homogeneity of the deposited films. The doped sols were then aged for 24 h at room temperature to complete the hydrolysis and condensation reactions and stabilize the system before deposition.

### 2.2 Preparation of Quinoline Derivative 4-(6-(diethylamino)-4- Phenylquinolin-2-yl)­benzoic Acid Solution

The QD (whose structure is shown in Figure 1) was synthesized according to a literature procedure,^24^ with minor modifications. First, the reaction was carried out on rather enlarged scale (5 mmol instead of 1 mmol). Second, there was a prior formation of the Schiff base before the addition of phenylacetylene, whereas in the original method the reaction was fully one-pot. This modification may favor the formation of the imine intermediate and reduce competing reactions, thereby improving control over the cycloaddition step. In addition, the reported temperature (130 °C) was higher than the typical reflux temperature of an aqueous HCl solution (∼100 °C), suggesting more energetic conditions that may accelerate the acid-catalyzed cyclization. However, the main difference lies in the purification procedure: instead of direct neutralization with NaOH followed by organic extraction, an acid–base workup using NaHCO_3_ and subsequent acidification was employed, taking advantage of the carboxylic acid group present in the product. This approach allows the quinoline derivative to be selectively transferred to the aqueous phase as its carboxylate salt and to precipitate after reacidification, resulting in greater selectivity and potentially higher final purity of the product. The characterization data for the obtained QD are reported in the Supporting Information file (figure S1).

**Figure 1:**
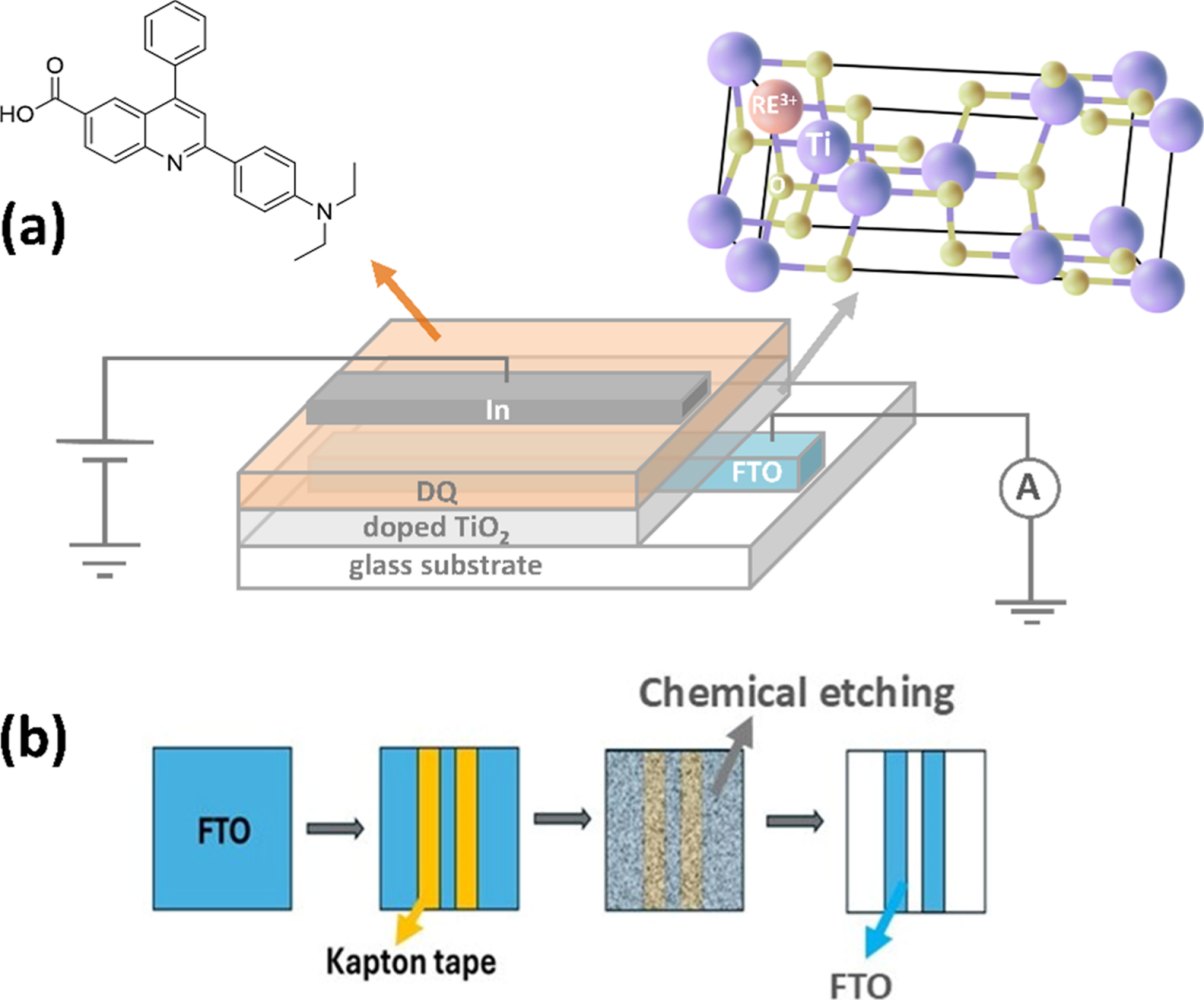
(a) Quinoline derivative (QD) 4-*(6-(diethylamino)-4-phenylquinolin-2-yl)­benzoic* structure (top left). Anatase structure of the TiO_2_ (top right) Layout for the heterostructure of TiO_2_ and QD thin films, deposited on glass with FTO channels and top In electrode (center). (b) Corrosion process of the FTO layer, showing a typical procedure with two contacts in the bottom of the deposited material.

Before thin-film deposition, the QD was dissolved in acetone at a concentration of 1 mg mL^–1^. Acetone was selected because of its good solubility with the compound and low boiling point, which facilitates homogeneous deposition. As an aprotic solvent, acetone can act as an electron-pair donor, aiding in the solvation of the QD.^25^ The use of other types of solvents has been reported elsewhere.^26^

### 2.3 Thin-Film Deposition and Heterostructure Sample Assembly

TiO_2_ thin films were deposited onto microscope glass substrates using the dip-coating method, by means of a MQB SG 1/302 dip-coating pump connected to a MQCTL2000-MP microprocessor controller. The substrates were immersed and withdrawn at a controlled rate of 10 cm·min^–1^, repeating the process three times to achieve the desired thickness. After each coating, the films were subjected to a gelation step (10 min), followed by an intermediate heat treatment at 110 °C for 10 min to promote densification and remove residual organics. After the final layer, the samples were annealed at 500 °C for 5 h in an EDGCOM 3P furnace for crystallization of mainly the anatase phase, whose structure can be visualized at the right inset of Figure 1a.

Table 1 presents the adopted codes for each sample with information about the percentage of doping with rare-earth ions Er^3+^ and Yb^3+^.

**Table 1:** Sample Codes and Percents Doped with Rare Earth Ions^a^

sample code	Er^3+^ doped (at %)	Yb^3+^ doped (at %)
undoped	0	0
TE2	2	0
TE4	4	0
TY2	0	2
TY4	0	4

a Placing the letter “H” in front of the acronym refers to heterostructure with the quinoline derivative. For instance, HTE2 means the heterostructure of QD and TiO_2_ doped with 2 atom % Er.

The deposition of the QD dissolved in acetone was performed on etched fluorine-doped tin oxide (FTO) and silica substrates using a Laurell Technologies spin coater. For each deposition, 0.5 mL of solution was dropped on the substrate 3000 rpm for 30 s under nitrogen atmosphere. After each of the five layers, films were heated to 70 °C for 5 min to evaporate the solvent, followed by a final annealing for 20 min. The total thickness was approximately 200 nm.

FTO was chosen due to its low sheet resistance (≈45 Ω/mm), which allows creating a channel for the deposition of the semiconductor material, where the central region of the FTO was chemically removed. To protect the desired FTO contact areas from corrosion, Kapton polyamide tape, resistant to high temperatures, was used and metallic zinc was inserted in the unwanted region, along with hydrochloric acid. Zinc acts as a catalyst for the chemical etching process, leading to total elimination of the FTO layer from region between contacts^27^ and beyond them, resulting in a well-defined contact geometry. This process is schematically shown in Figure 1b, exemplifying the case of two contacts in the bottom, designed for electrical characterization of individual QD or TiO_2_ films.

For the deposition of the heterostructures, thin films of TiO_2_ and of the QD were deposited onto borosilicate glass substrates also coated with FTO (fluorine-doped tin oxide), using a similar procedure of deposition of QD films. The TiO_2_ layers were prepared by the dip-coating method, while the quinoline-based organic layers were deposited by spin coating.

For the TiO_2_ layer, the deposition is similar of the individual films, being carried out by dip-coating of 7 layers. After each deposition, the films were subjected to the same intermediate thermal treatment at 110 °C for 30 min and the samples underwent a final annealing at 500 °C for 1 h, leading to the anatase phase.

The heterostructures combining TiO_2_ and QD layers are schematically shown in Figure 1a, along with the bias setup for electroluminescence measurement. The bottom layer followed the same deposition procedure as the individual TiO_2_ films, and the QD layer was spin-coated on top. The design of the device included a single bottom FTO contact and an evaporated indium (In) top electrode, allowing charge transport through both materials and their interface. This configuration enables the evaluation of the p–n junction behavior and electroluminescence measurement.

### 2.4 Sample Characterization

The structural properties of the TiO_2_ films were analyzed by X-ray diffraction (XRD), carried out on a Rigaku D/MAX–2100/PC using Cu radiation (Kα = 1.5405 Å) and Ni filter to attenuate the Kβ radiation. The scan was performed at a rate of 2°/min, in the range 20–60°. The diffraction patterns were used to identify the crystalline phase and to evaluate the possible influence of rare-earth doping on the structural organization of the TiO_2_ matrix. The average crystallite size was estimated by using the Scherrer equation.^28^

The chemical bonding environment and possible interactions at the organic–inorganic interface were investigated by Fourier-transform infrared spectroscopy (FTIR). Special attention was given to vibrational modes associated with the QD and to bands related to lattice oxygen and defect sites in TiO_2_, allowing the assessment of molecular coordination and ligand–surface interactions.

Surface morphology and film uniformity were examined by confocal optical microscopy (Leica system), enabling direct visualization of topographical features and layer coverage. Scanning electron microscopy (SEM) (TÜVRheinland) provided higher-resolution images of surface texture and grain distribution. To assess dopant incorporation and spatial homogeneity, energy-dispersive X-ray spectroscopy (EDS) mapping was performed in multiple regions across the TiO_2_:RE^3+^ films.

Optical absorption spectra were collected using a Cary 5000 UV–Vis spectrophotometer to evaluate electronic transitions and estimate the optical bandgap of the materials. Photoluminescence (PL) spectroscopy was used to probe the emission characteristics of the hybrid heterostructures, assessing the role of rare-earth doping in modifying radiative recombination processes.

For electroluminescence (EL) measurements, the hybrid structures were electrically biased using a Blausonic electrometer, with negative polarity applied to the TiO_2_ layer and positive polarity to the QD. This configuration allows charge injection across the heterointerface, enabling the evaluation of light emission under electrical excitation and confirming the formation of an active p–n junction.

The experimental setup used to perform all photoluminescence measurements is shown in Figure 2, which consisted of a 445 nm diode laser (FC–445–2W) as the excitation source, whose optical signal was directed to an optical fiber coupled to a collimator (1), and at a distance of 50 mm, a converging lens with a focal length of 50 mm was positioned (2). Next, at a distance of 150 mm, another lens with a focal length of 100 mm was placed (3). Subsequently, 150 mm from this, another lens with a focal length of 50 mm was positioned (4), located 50 mm from the sample (5), which in turn was linked through an optical fiber channel (placed to collect the luminescence at 90°) to a Yokogawa optical spectrum analyzer (6).

**Figure 2:**
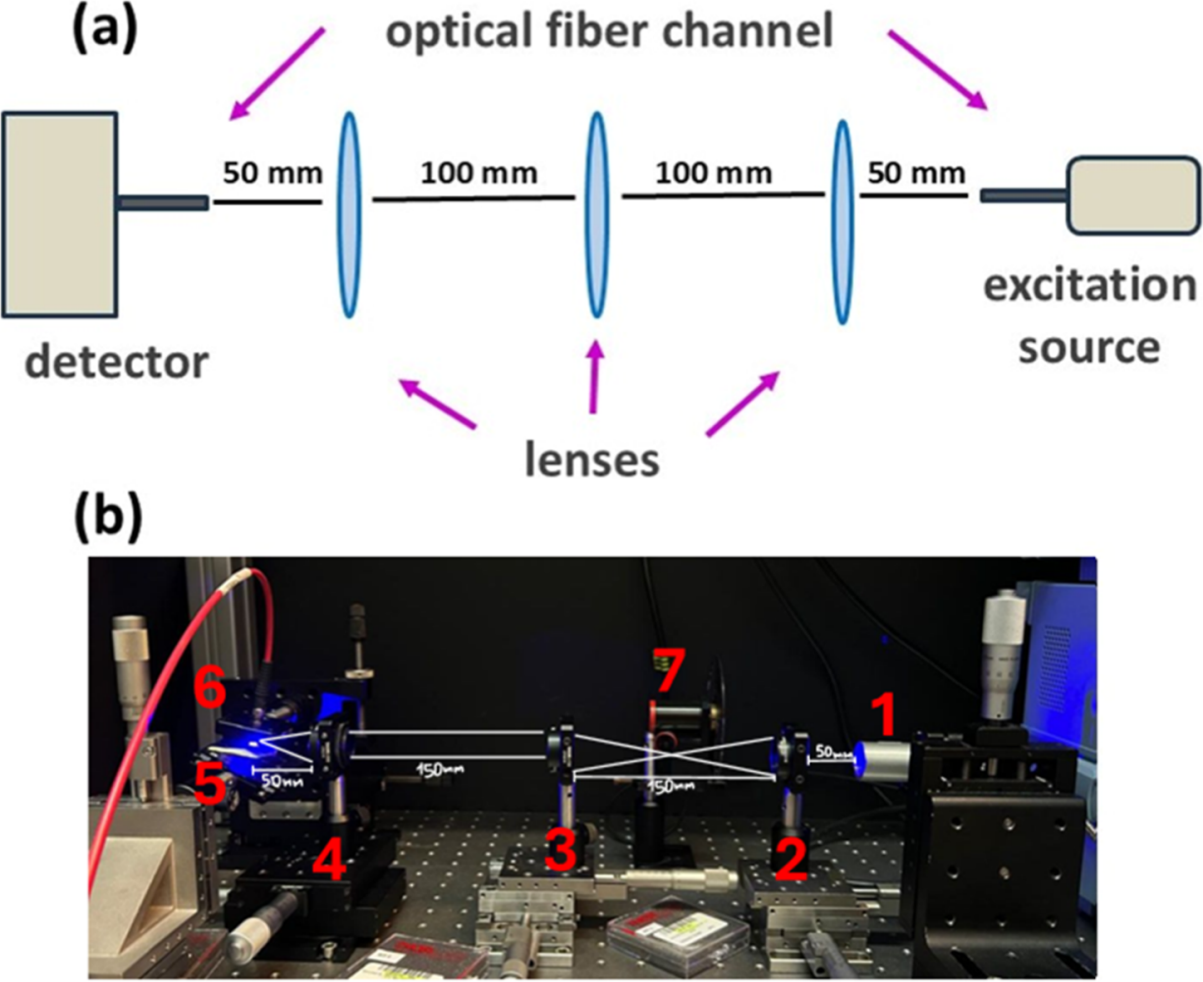
(a) Setup of photoluminescence measurements. (b) Image indicating the equipment disposition.

## 3 Results and Discussion

### 3.1 Structural and Optical Characterization of Lanthanide-Doped TiO_2_

Figure 3 presents X-ray diffractogram data (left-Figure 3a) and the absorbance spectra of TiO_2_ samples, with distinct doping levels (center-Figure 3b and right-Figure 3c). The X-ray diffractogram shows the anatase typical profile (COD database ID 1010942) and allows inferring that the doping process induced slight structural disorder, evidenced by the broadening of the peaks and a decrease in intensity. The absorbance spectra aim to identify the absorption bands corresponding to each doping level. It is well-known that doping with rare-earth ions inhibits crystallite growth and leads to smaller crystallites,^29,30^ and it is related to the replacement of Ti^4+^ ions by Er^3+^ or Yb^3+^, which, due to their size difference, can cause lattice distortions. Besides, the doping excess migrates toward the grain boundary layer inhibiting the crystallite growth. Furthermore, due to the difference in charges, it is expected that doping induces oxygen vacancies to balance it, leading to some disorder.^31^ No new peaks show up in the diffractogram, which indicates that the doping was incorporated into the TiO_2_ matrix lattice without the generation of distinct phases such as erbium or ytterbium oxide.

**Figure 3:**
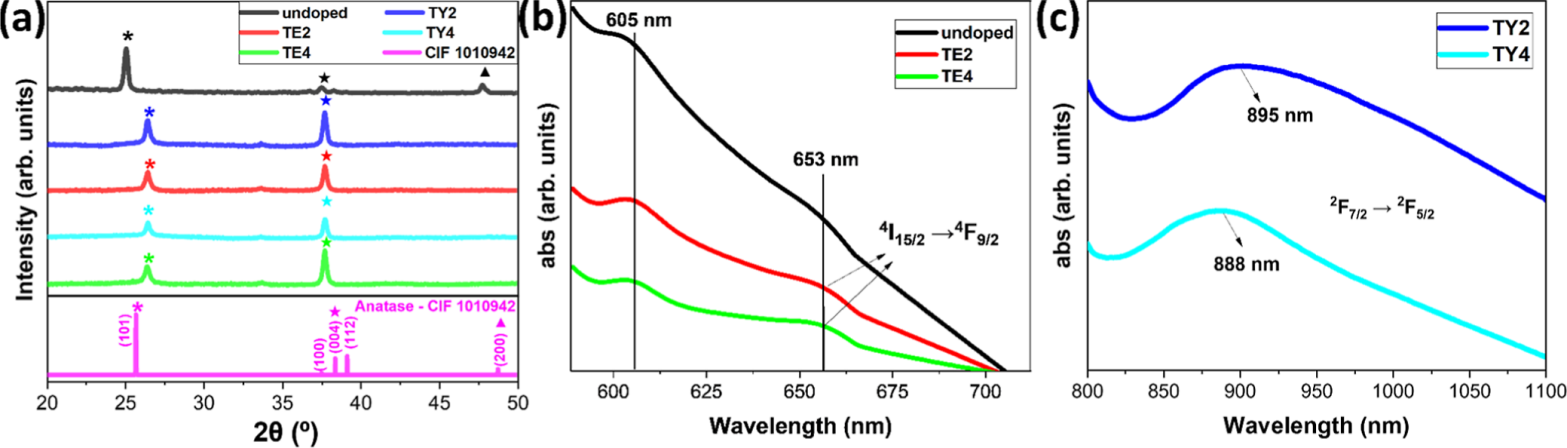
(**a**) X-ray diffractogram. **(b)** Absorbance of TiO_2_ thin films undoped and doped with Er^3+^ **(c)** doped with Yb^3+^.

From optical absorption data, bandgap values have been calculated according to the Tauc Plot,^32^ for indirect bandgap transition, which is the case of anatase structure. The Tauc plot is presented in Figure 4, and bandgap values are listed in Table 2. The Tauc plots exhibit a clear dependence on the film morphology, as evidenced by the SEM analysis (Figure 5, below and Figures S2 to S4 of Supporting Information). The undoped sample, which presents a more homogeneous and compact microstructure, shows the lowest bandgap value of 2.95. eV, consistent with a reduced contribution from structural disorder and less light scattering effects, thus more closely reflecting the intrinsic electronic structure of TiO_2_, with a value closer to the literature for anatase.^32^ Upon doping, significant morphological changes are observed, including increased porosity and surface irregularities, particularly in the Er-doped TiO_2_ sample, TE2, with a higher degree of porosity compared to that of TE4. This increased structural disorder induces more light scattering and affects the bandgap value, being as high as 3.27 eV for the TE2 sample. For Yb-doped TiO_2_ samples, the effect is even more pronounced. SEM images (figures of the Supporting Information) reveal a highly heterogeneous morphology with the presence of large agglomerates and nonuniform surface features, which is expected to increase both light scattering and local structural disorder. Then, the fundamental optical absorption edge may be affected, and then, the simple linear extrapolation in the Tauc analysis leads to values of about 3.75 eV. Thus, the overall influence of doping on the bandgap transition is attributed not only to morphological effects and light scattering but also to disorder-induced modifications in the electronic structure, including band tailing and possible changes in the dominant optical transitions.

**Figure 4:**
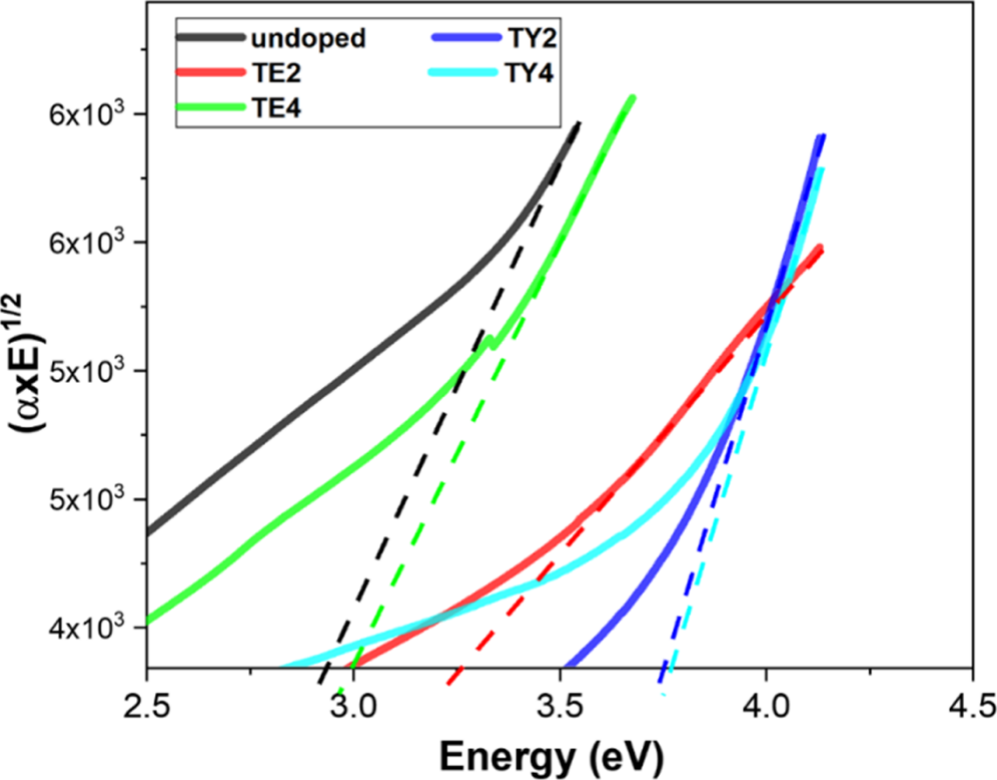
Tauc Plot for indirect bandgap transition of TiO_2_.

**Table 2:** Bandgap Evaluation from Optical Absorption Data, Using the Tauc Plot

sample	bandgap values (eV)
undoped	2.95
TE2	3.27
TE4	3.00
TY2	3.75
TY4	3.76

**Figure 5:**
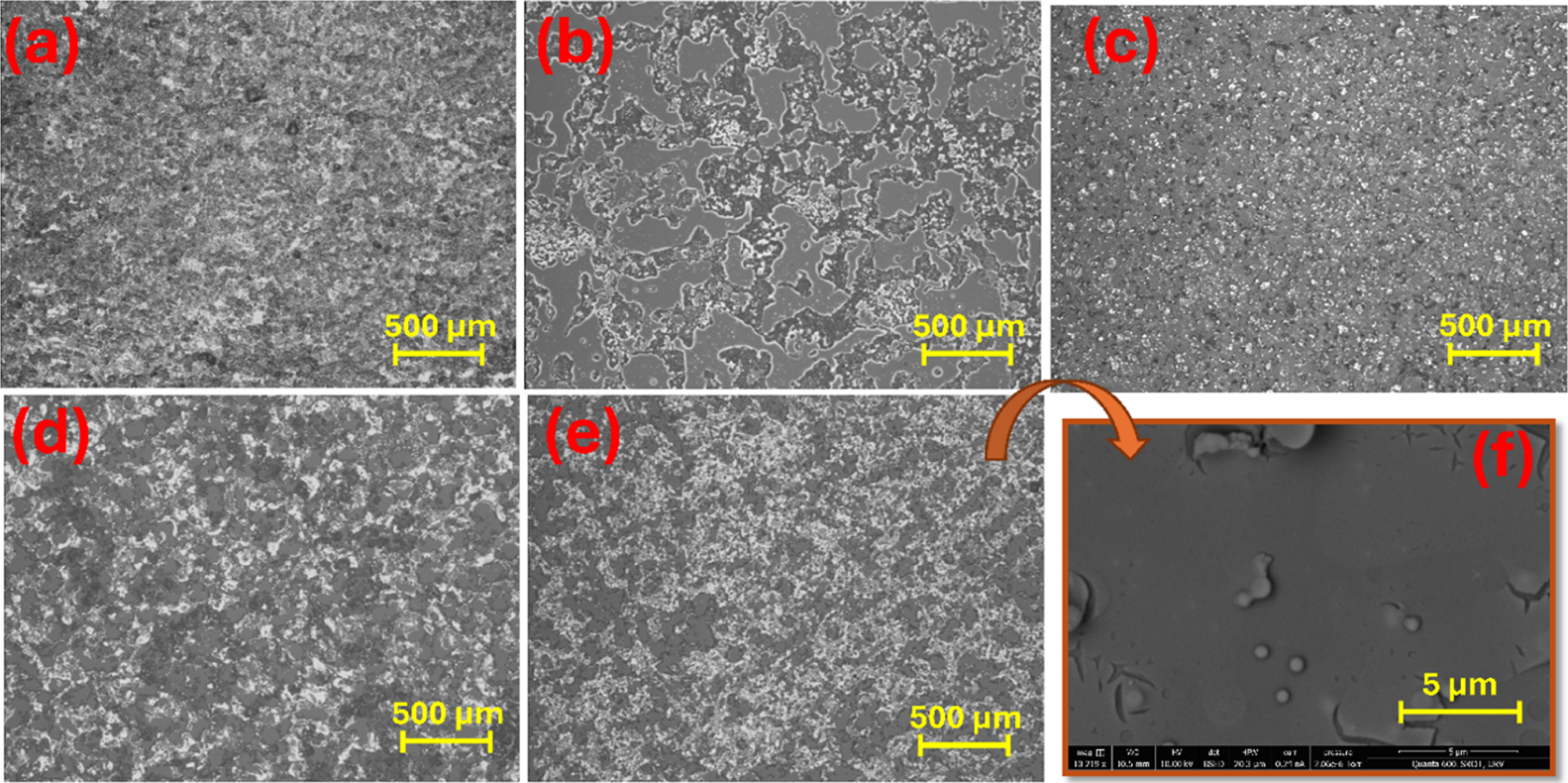
Confocal microscopy images of TiO_2_ films doped with (**a**) 2% Er^3+^, (**b**) 4% Er^3+^, (**c**) 2% Yb^3+^, (**d**) 4% Yb^3+^, (**e**) undoped. (**f**) SEM images of 4% Er^3+^: TiO_2_, magnification: 10,000×.

Due to the creation of defects by the insertion of trivalent ions into the structure, it is also possible to observe a slight shift of the peaks to a higher 2θ angle,^33^ compared to undoped TiO_2_. It is also interesting to notice that the peak associated with the plane (004) has increased intensity and, in some cases, becomes even higher than the peak due to plane (101), which is the main in the anatase TiO_2_ structure.^34^ Then, the analysis of the texture coefficient indicated a preferential orientation in the (004) direction for all the doped samples, which increases with higher doping. This evaluation is shown in the Supporting Information file (Table S1). In anatase TiO_2_, crystallographic orientation strongly influences surface energy^35^ and the density of coordinatively unsaturated Ti sites, which govern molecular anchoring and interfacial electronic structure, being very relevant for heterostructures, as proposed in the present work.

The average crystallite size was calculated using the Scherrer equation^28^ for the plane (101) of the anatase TiO_2_ structure, yielding the values 24.1, 20.4, 21.6, and 18.9 for samples TE2, TE4, TY2, and TY4, respectively. As expected, as the doping concentration increases, the average crystallite size decreases.

The doping of TiO_2_ with Er^3+^ and Yb^3+^ ions did not promote a significant variation in the crystallite size in the plane (101) when compared to the undoped sample, in which an average crystallite size of 23.4 nm was obtained. A similar result has been reported,^36^ using rare-earth Er^3+^ and Eu^3+^ as doping ions, in which no noticeable shifts or broadenings of the diffraction peaks or relevant structural changes were observed, indicating that the introduction of dopants did not appreciably alter the crystallinity or the crystallite growth of TiO_2_.

This behavior suggests that at the investigated concentrations, the insertion of dopant ions was not enough to generate lattice strains capable of limiting crystalline growth. Furthermore, the applied thermal annealing, which was restricted by the thermal stability of the glass substrate, resulted in crystallites of similar dimensions, regardless of the presence of the dopant. Thus, doping likely introduces localized electronic states within the band gap, as suggested by defect-related absorption features observed in the UV–vis spectra (Figure 3b,c), without significantly impacting the surface energy involved in grain growth.

UV–vis absorbance measurements shown in Figure 3b,c display weak but well-defined features associated with intra4f electronic transitions of Er^3+^ and Yb^3+^ ions, as well as contributions attributed to structural defects, which are consistent with XRD data, allowing a direct correlation between optical absorption and structural defects. At 605 nm, there is a band related to defects in the TiO_2_ matrix, as they are present in all spectra, including the data for the undoped sample, and their intensity can increase depending on the doping concentration. It is also possible to observe the characteristic transition of Er^3+^ ion ^4^I_15/2_ → ^4^F_9/2_ at 653 nm.^37^ In the case of samples doped with Yb^3+^, a broad absorption band was observed in the IR region of 800–1000 nm, which corresponds to the only absorption transition of this ion (^2^F_7/2_ → ^2^F_5/2_).^38^ However, such an unusually broad absorption feature is not typically observed for rare-earth ions. This behavior can be attributed to inhomogeneous broadening effects associated with structural disorder. In disordered rare-earth-doped systems, variations in the local crystal field surrounding the ions lead to a distribution of energy levels, resulting in broadened optical transitions.^39^

In the present case, the sol–gel-derived TiO_2_ matrix, combined with the incorporation of Yb^3+^ ions, promotes structural disorder and the formation of defect states. This leads to a distribution of local crystal-field environments and, consequently, to the observed broad absorption band. This interpretation is further supported by SEM analysis (as seen in Figure 5e, below, and Figures S2 to S4 of the Supporting Information), which reveals a more heterogeneous and less uniform morphology for Yb^3+^-doped films compared to Er^3+^-doped samples, indicating a higher degree of structural disorder.

Figure 5 shows confocal microscopy images of undoped samples and samples doped with 2 atom % and 4 atom % rare earth ions Er^3+^ and Yb^3+^. It is easily seen that doping causes modifications in the film surface morphology. Undoped TiO_2_ (Figure 5e) presents a relatively homogeneous surface, with a granular morphology typical of films obtained by solution deposition methods. With the addition of rare-earth doping, the surface aspect changes significantly, revealing more porous regions and a reduction in apparent granularity. This morphological alteration suggests that dopants directly influence the crystallization and grain growth processes, promoting local variations in surface energy that result in changes in particle packing.^40^ The Er^3+^ ion leads to more porous and less dense structures (Figure 5a,b) compared to the undoped film as previously reported.^41^ The shown images allow us to observe that the presence of Ln^3+^ ions in general reduces the nanoparticles size and avoids aggregation, leading to a more uniform distribution, although disperse. This adherence inhibition prevents a denser matrix, favoring an open microstructure, with higher surface area and higher porosity. Early reports^42^ show that at low concentrations the grains are approximately spheric and linked to each other, however with voids, in good agreement with the observed here (Figure 5c). When the Yb concentration increases, there is progressive reduction of these voids and a more uniform and compact structure occurs (Figure 5d), with denser and aggregated grains and less definite boundaries. This effect can be associated with Yb^3+^ as the modifier, since its incorporation into the oxide lattice generates local stress, modifying the surface energy of crystalline planes and changes the nucleation rate and grain growth. Then, a higher concentration of Yb^3+^ favors more homogeneous growth and decreases surface imperfections whereas lower concentration maintains incomplete coalescence and morphological voids as observed. Figure 5f shows SEM images for TiO_2_ films doped with 4% Er^3+^. SEM images for samples with 2% Er and 2% and 4%Yb are shown in the Supporting Information file (figures S2 to S4). In this higher magnification (Figure 5f), compared to confocal image, the surface seems rather homogeneous even though some agglomerates are starting to become visible. Based on the SEM images, average particle size for Yb-doped TiO_2_ was evaluated and yields 2.95 μm for 2 atom %Yb (TY2) to 2.65 μm for 4 atom % Yb (TY4). The distribution of particle size is shown in the Supporting Information file (Figure S5). The incorporation of ions in the TiO_2_ lattice induces lattice distortion along with localized distortions in the lattice, decreasing the grain size for a higher doping level. On the other hand, for Er-doped TiO_2_ it is possible to verify higher porosity for the lower doping (TE2) and a more homogeneous surface for sample TE4.

Figure 6 shows atomic concentration maps (scan EDS) of titanium, oxygen, and the respective rare-earth doping. For Er^3+^-doped samples, EDS measurements indicated that the doping atomic percentage did not fluctuate significantly among the different regions of the surface. In contrast, for Yb^3+^-doped samples, a rather more heterogeneous doping distribution was observed. SEM images and scan EDX for 2 atom % Er and 2 atom % Yb can be found in the Supporting Information (Figures S2 and S3).

**Figure 6:**
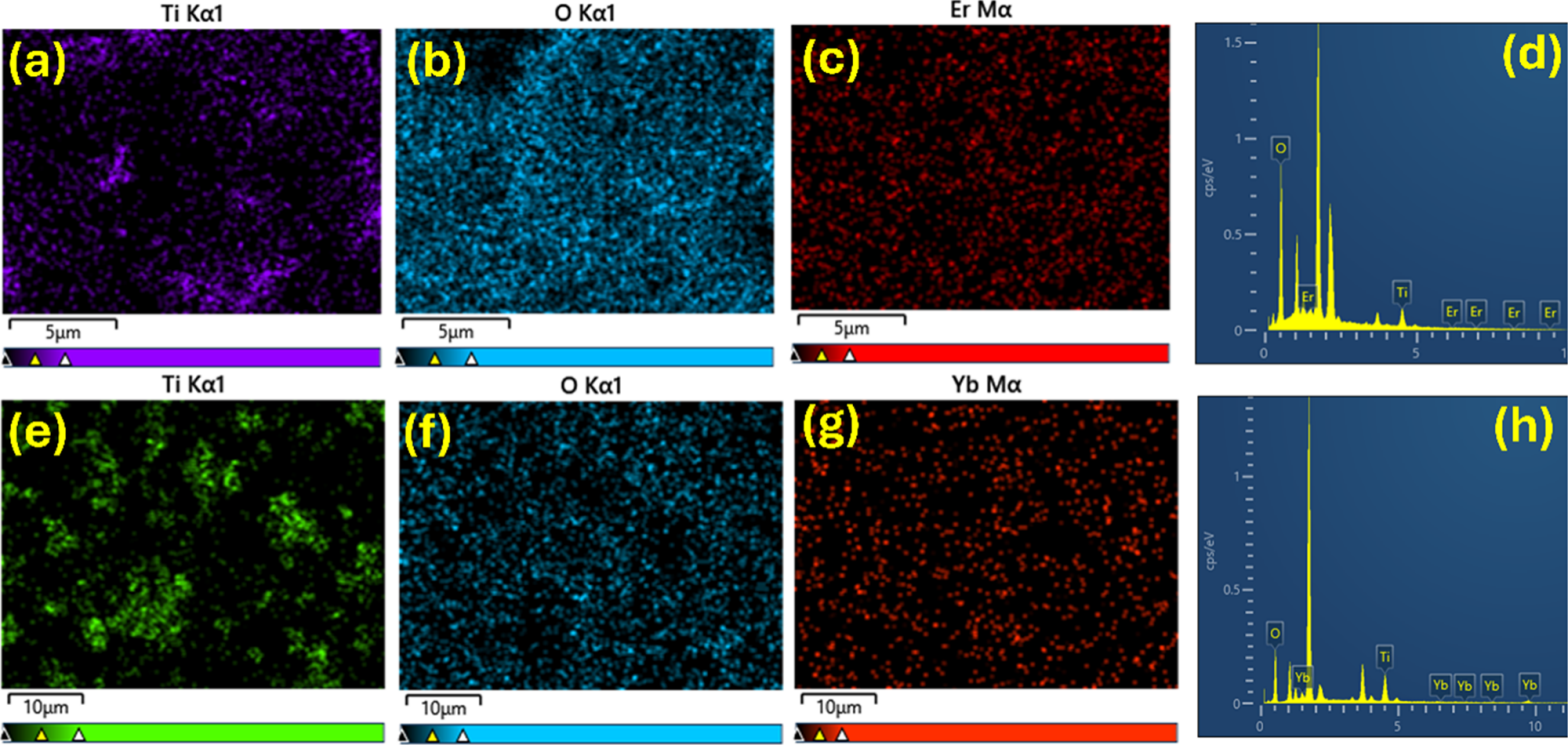
Scan EDX for 4% Er^3+^: TiO_2_ (**a**) Titanium, (**b**) Oxygen, (**c**) Er, (**d**) EDX data and scan EDX for 4% Yb^3+^: TiO_2_ (**e**) Titanium, (**f**) Oxygen, (**g**) Yb, (**h**) EDX data.

### 3.2 Quinoline Derivative: Morphology, Optical and Electrical Characterization

Figure 7a brings confocal microscopy images of the QD film, showing some peculiarities on the surface similar to dendritic patterns, and Figure 7b shows the absorbance spectra of a QD thin film. The formation of dendritic patterns in organic thin films, as seen in Figure 7a images, has been widely associated with phase separation mechanisms, crystallization, and electrostatic effects during solution deposition or drying. Haberko et al.^43^ reported similar dendritic formation in mixtures containing doped polyaniline and insulating polymers such as PS or PMMA, obtained by spin-coating.

**Figure 7:**
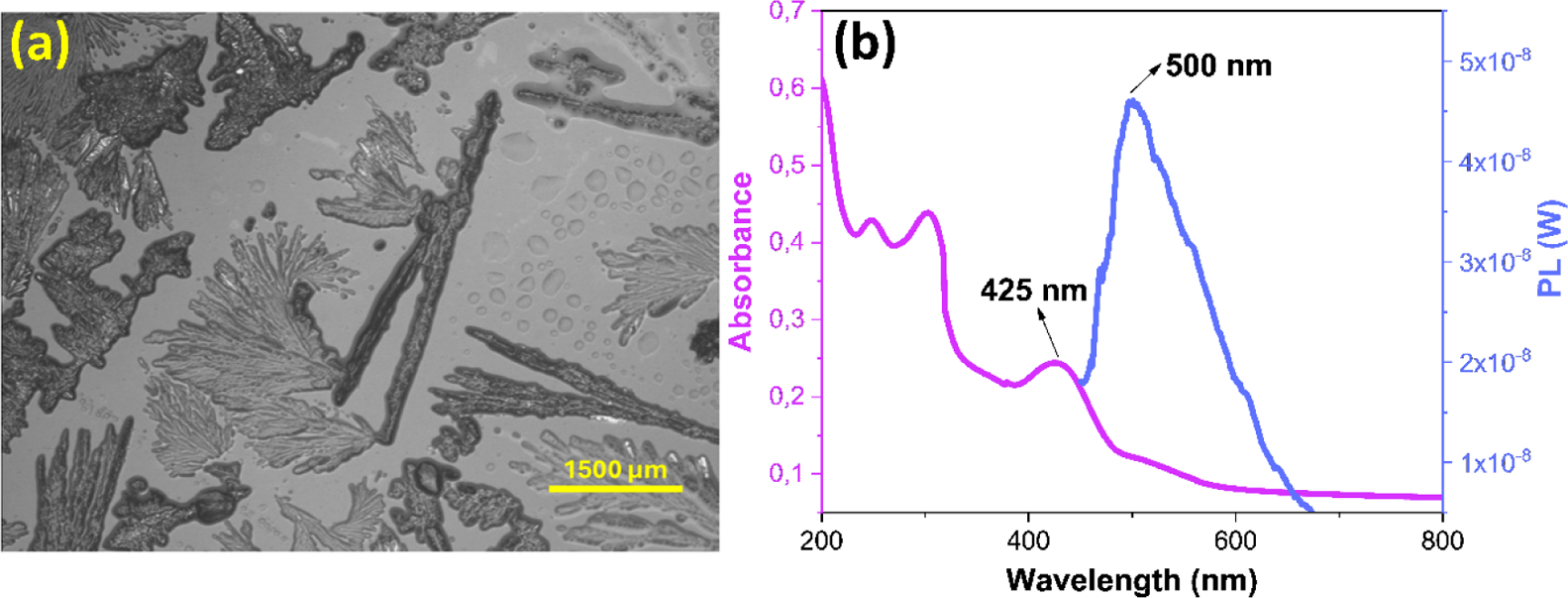
Quinoline derivative data. (**a**) Confocal microscopy images and (**b**) absorbance and photoluminescence of an individual film.

Complementarily, Gao et al.^44^ and Juang et al.^45^ showed that films of *7,7,8,8-tetracyanoquinodimethane* deposited by an ionized cluster beam can exhibit chiral S-shaped dendrites, resulting from the interaction between diffusion and local electrostatic fields generated by charge accumulation during growth. These authors demonstrated that chirality and the final morphology result from spontaneous symmetry breaking in the presence of internal electric fields, which direct the growth of branches. Thus, the literature indicates that the formation of dendritic structures in organic materials can occur through different mechanisms, ranging from electrostatic instabilities to phase separation induced by concentration gradients, all capable of generating hierarchical self-organization similar to that observed in the single film of the quinoline derivative in Figure 7a.

The absorbance spectra of a QD thin film shown in Figure 7b display the main absorption bands, which are rather wide, peaking at 247, 300, and 425 nm, and the shown photoluminescence band peaking at 500 nm is rather wide as well. The absorptions in the blue range of the electromagnetic spectrum are due to the conjugated double bonds present in the structure of this QD and are attributed to molecular orbital transitions.^46^ The Stokes shift, calculated from the difference between the blue absorption maximum (425 nm) and the PL maximum (500 nm), is approximately 75 nm (0.44 eV), indicating significant excited-state relaxation prior to radiative recombination.

The D–π–A molecule (QD) used here has a relatively large Stokes shift and the broad emission band centered at 500 nm, which are consistent with substantial excited state reorganization and partial charge-transfer character of the radiative emitting state.^19,20^ These features suggest that emission in the isolated film originates from an intramolecular charge transfer (ICT) state stabilized by structural and electronic redistribution upon excitation.

As will be seen, upon integration with TiO_2_ forming a heterostructure, the emission maximum shifts to 483 nm, evidencing a modulation of the excited-state electronic structure at the interface. This blue shift indicates a reduction in ICT stabilization, likely arising from interfacial electronic coupling and changes in the local electrostatic environment.^47^ These findings demonstrate that the emissive state is not purely intrinsic to the organic layer but is significantly influenced by the interfacial interactions within the heterostructure.

Figure 8 brings current as a function of time for optical excitation of a QD derivative film with an InGaN LED that presents an average emission wavelength at 450 nm and an average power of 15 mW. The figure displays current using this excitation, and the decay after the irradiation is removed. The inset in Figure 8 is a fitting procedure of the decay portion using a single-exponential decay approach.

**Figure 8:**
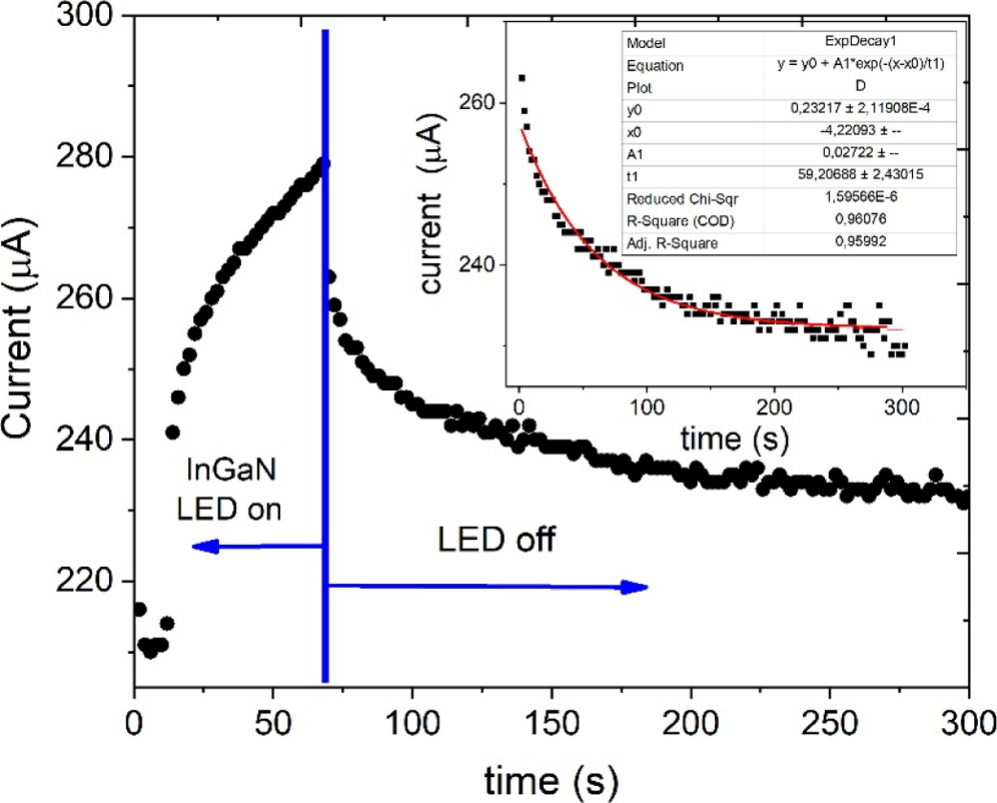
Current as a function of time for excitation with a InGaN LED (450 nm), and decay after irradiation removal. ***Inset***: exponential data fitting for the decay part.

The current increase upon excitation observed in Figure 8 is related to the blue absorption band shown in Figure 7. Although this band has a peak about 428 nm, the LED is a broad source and has intensity enough to excite this band. The decay observed in Figure 8 can be modeled considering the dominant transitions and localized defects. The trapping effects concomitant with molecular structure relaxation^48^ may be responsible for the exponential like excitation and decay. Despite the rather long curve in Figure 8 upon excitation (about 70 s), it does not lead to complete saturation yet. The decay data fitting can be approximated to a function as eq 1.

1 $$I=Io+Ic.\exp\left(-\frac{t}{to}\right)$$

where *I*_*o*_ is the background current (dark value), *I*_*c*_ is the maximum variation in the excited current, and the constant *t*_*o*_ is the characteristic time. In the present case, *t*_*o*_ is equal to ∼59.2 s, which means a decay of approximately 37% (e^–1^ factor) of the initial excited current (given by Io + Ic ≈ 279 μA) and it is related to the dark steady state value (about 232 μA), which can be considered rather long and may be associated with molecular structure relaxation on the carrier trapping by defects. A photoinduced state of a defect represents a metastable state, which can be separated from the ground state by a potential barrier. This situation can be represented in a configurational energy diagram,^48,49^ where the potential barrier in general represents the molecular structure relaxation around the defect when the photoinduced carrier is trapped back. Considering that the main conduction mechanism in these compounds is VRH,^22^ the existence of a characteristic relaxation time is in good agreement with this mechanism. Liu et al.^50^ observed a charge carrier lifetime of about 2 h through engineering of the interface band structure coupling, controlling the spatial overlap of the photoexcited electron and hole populations and the recombination rate of the device. Although the present measurements were performed on individual quinoline derivative films, the observation of long-lived photoinduced states is optically relevant, since the light-emitting layer governs exciton formation and recombination under electrical operation. Such metastable states may also be relevant for emission and electronic transport layers in LEDs, as they can influence charge transport and carrier persistence under continuous excitation.

### 3.3 Heterostructure Characterization

Figure 9 shows FTIR results for undoped TiO_2_ films, TiO_2_ doped with Er^3+^ and Yb^3+^, QD films, and data of heterostructures. The inset in Figure 9b is a representation of the proposed bidentate coordination mode of QD at the TiO_2_ surface. In-plane and out-of-plane aromatic C–H deformation modes are observed in the 1300–850 cm^–1^ and 950–600 cm^–1^ ranges, respectively, consistent with quinoline-based systems.^51^ These bands remain present in the heterostructures, indicating the preservation of the molecular backbone after integration with TiO_2_. On the other hand, for the TiO_2_ films, the spectral region between 400 and 900 cm^–1^ is dominated by Ti–O–Ti lattice vibrations, characteristic of partially crystalline oxide phases. The broad features observed in this range reflect the structural disorder typical of sol–gel-derived TiO_2_ films.^51^

**Figure 9:**
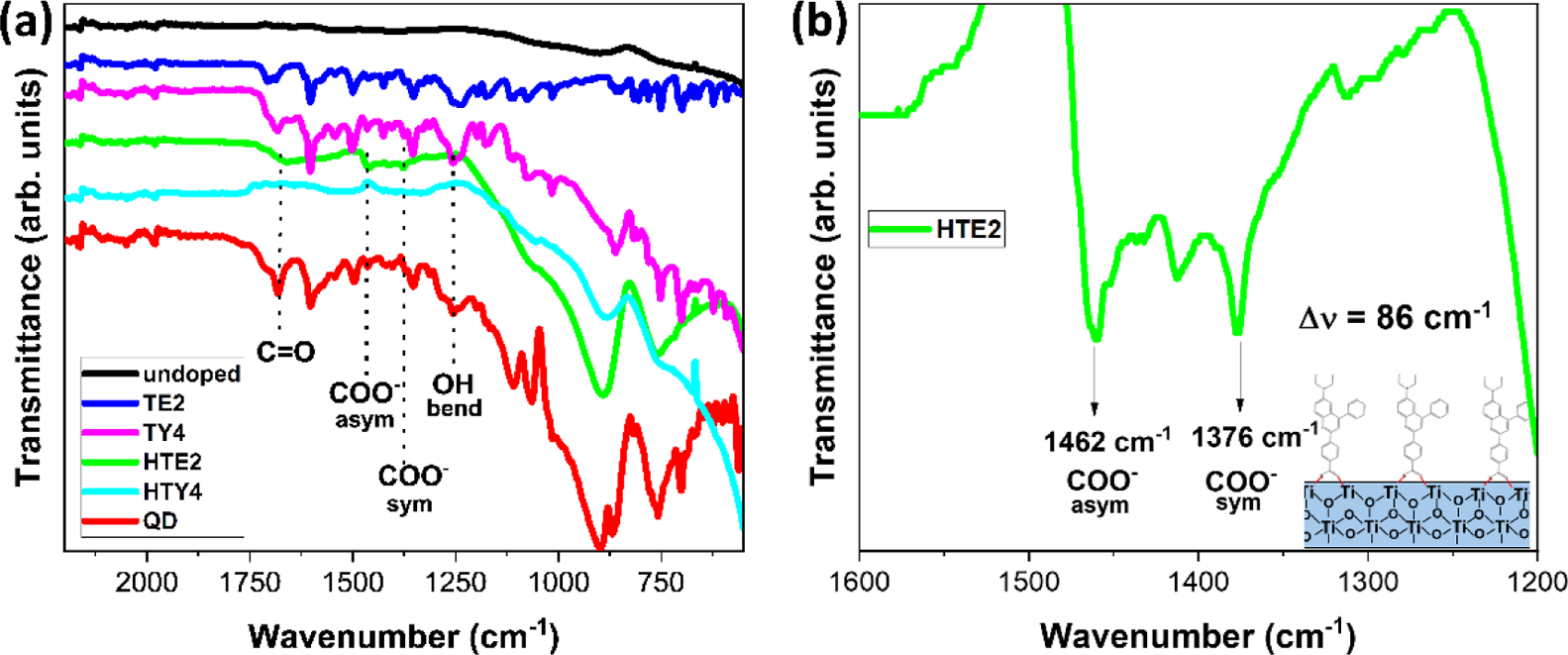
(a) FTIR spectra for TiO_2_ films and heterostructure samples. (**b**) FTIR spectra of the HTE2 for a shorter range, highlighting the stretching modes of the COO^–^. ***Inset:*** Schematic representation of the proposed bidentate coordination mode at the TiO_2_/QD interface.

The characteristic C=O stretching band (1684 cm^–1^), observed in the isolated QD, disappears after the formation of heterostructures with TiO_2_ for both the Er^3+^- and Yb^3+^-doped systems. This behavior indicates modification of the carboxyl group of the molecule, suggesting deprotonation.^52,53^ In the HTE2 hybrid system, the observed distinct bands are attributed to the asymmetric (∼1462 cm^–1^) and symmetric (∼1376 cm^–1^) stretching modes of the carboxylate group (COO^–^), which are not observed in individual film QD spectrum. The frequency difference between the asymmetric and symmetric modes (Δν ≈ 86 cm^–1^) is consistent with a bridging bidentate coordination to the TiO_2_ surface, as illustrated in the inset of Figure 9b, indicating an organized chemical anchoring and strong interfacial coupling.^54,55^ Such anchoring is expected to promote a defined molecular orientation relative to the TiO_2_ surface and to facilitate efficient electronic coupling at the organic–inorganic interface.^56^ This bonding configuration is reported to be energetically favorable, exhibiting higher binding energies according to calculations based on the density functional theory (DFT).^57^ It may be related to the coordination of electron donor groups of the QD organic molecule with surface Ti^4+^ centers and/or oxygen vacancies present in the lattice.^58^ Such coordination suggests that Er^3+^ doping promotes a more reactive TiO_2_ surface, exhibiting significant vacancy concentration, and, consequently, the formation of stronger interfacial bonds. Similar behavior was reported by Singha et al.,^59^ who observed the shift and attenuation of FTIR bands in heterostructures.

In contrast, although the C=O band also disappears in the HTY4 system, no well-defined characteristic bands of the COO^–^ species are observed. This behavior shows that there was a chemical modification of the carboxylic acid group, but the absence of vibrations related to COO^–^ suggests a more heterogeneous interface, in which the interaction with the TiO_2_ surface may follow a distinct coordination mode.

From an atomic-scale perspective, the substitution of Ti^4+^ by Ln^3+^ ions introduces a charge imbalance in the TiO_2_ lattice, which is typically compensated by the formation of oxygen vacancies.^60^ These defects generate undercoordinated Ti sites and localized electronic states.

Upon interaction with the TiO_2_ surface, the carboxylic acid group of the quinoline derivative undergoes deprotonation, leading to the formation of a carboxylate group (COO^–^). This group can coordinate with surface Ti atoms, and the bridging bidentate configuration is energetically favored because it coordinates to two Ti atoms on the surface of TiO_2_, forming a more stable interaction than the monodentate one.^52^ Furthermore, the more homogeneous dopant distribution observed in SEM images for Er^3+^ leads to a more uniform surface, which may lead to well-defined reactive sites, favoring organized coordination. In contrast, the heterogeneous distribution in Yb^3+^-doped samples may result in less-defined coordination modes.

The more defined bidentate coordination observed for the HTE2 system likely reduces the interfacial injection barrier,^55^ explaining the lower turn-on voltage observed in the electroluminescence data, shown in the next section. Conversely, the absence of well-defined carboxylate modes in the HTY4 system suggests a more heterogeneous interface, which may contribute to improved emission under high electric fields by mitigating the localized charge accumulation.

XPS measurements on the TE2 sample are shown in Figure 10, which were performed to investigate the surface chemical environment of the doped TiO_2_ films. The spectra were deconvoluted with CasaXPS software. The Ti 2p spectra confirm that titanium is predominantly present as Ti^4+^, indicating that the TiO_2_ matrix preserves its expected oxidation state without evidence of Ti^3+^ formation. This suggests that the electronic structure is mainly influenced by other defect states rather than the reduction of Ti centers.

**Figure 10:**
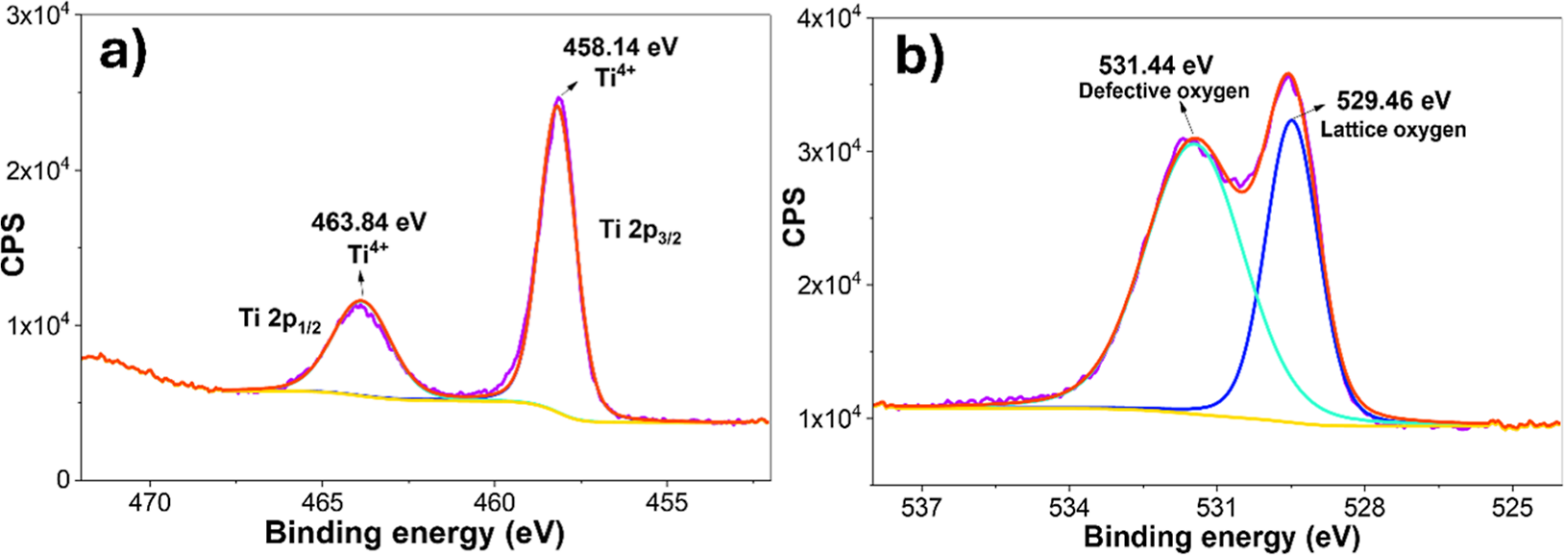
XPS spectra; (a) Ti 2p (b) O 1s.

The O 1s spectra show two different peaks, which were deconvoluted into distinct components. The peak at 529.46 eV represents lattice oxygen with 37.52% of the total area, and the peak at 531.44 eV is associated with defective oxygen, and it represents 62.48% of the total area, revealing a significant contribution associated with defect-related oxygen species, commonly attributed to oxygen vacancies or nonlattice oxygen.^61^ The presence of these defect states is consistent with the substitution of Ti^4+^ by Er^3+^ ions, which requires charge compensation and promotes the formation of oxygen vacancies.

It has been reported that for stoichiometric and structurally unmodified TiO_2_, the high-resolution O 1s XPS spectrum is typically described as a single, symmetric peak predominantly associated with lattice oxygen.^62^ In such cases, contributions from defective oxygen species, vacancies, and surface groups are small. In contrast, the spectrum obtained in this work clearly exhibits two well-defined components, indicating the presence of distinct chemical environments for oxygen, possibly associated with structural defects and/or adsorbed surface species. XPS analysis provides support for the proposed interfacial coupling mechanism. The oxygen vacancies and Ti^4+^ surface sites act as active centers for coordination with the carboxylate group of the quinoline derivative, facilitating the formation of interfacial bonds as suggested by the FTIR analysis (Figure 9). Therefore, Figure 10 reinforces the interpretation that Ln^3+^ doping not only modifies the bulk properties of TiO_2_ but also enhances the surface reactivity, promoting stronger chemical anchoring and improved electronic coupling at the TiO_2_/QD interface.

**Figure 11:**
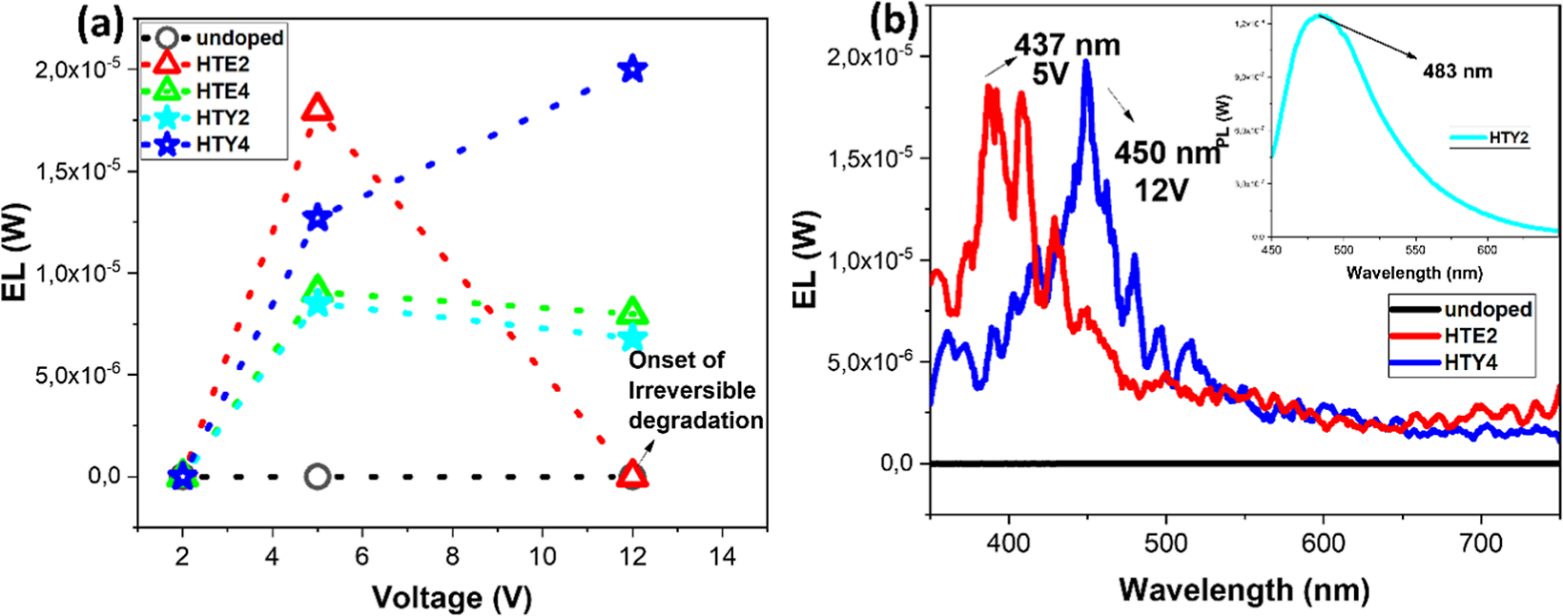
(a) Electroluminescence intensity peak as a function of the applied voltage. (**b**) Electroluminescence of the heterostructures with the highest efficiency. ***Inset***: Photoluminescence of the heterostructure HTY2.

### 3.4 Electroluminescence of the Heterostructure

Electroluminescence (EL) measurements were also carried out on the heterostructures, according to the procedure described in the experimental section, and applying a negative bias to the TiO_2_ layers and a positive bias to the QD layer. The emitted color was collected with the same photoluminescence system in a dark environment. The most marked results are shown in Figure 11, which shows the electroluminescence intensity peak as a function of the applied voltage for heterostructure samples (Figure 11a) and electroluminescence spectra of the heterostructures with the highest efficiency (Figure 11b). The inset in Figure 11b is the photoluminescence spectra of the heterostructure HTY2.

Results displayed in Figure 11 revealed that heterojunctions TiO_2_/QD perform as light-emitting diodes (LEDs) when the TiO_2_ layer is doped with rare-earth ions, besides exhibiting diode-like behavior as previously reported.^63^ The lanthanide ions introduce electronic levels within the TiO_2_ layer band gap, acting as radiative recombination centers that facilitate light emission. This effect is consistent with that observed in double perovskites doped with lanthanide trivalent ions such as Dy^3+^, Eu^3+^, and Sm^3+^,^64^ in which the doping modifies the local crystal field and promotes the formation of oxygen vacancies, resulting in greater electron–hole recombination efficiency and intensified photoluminescence.

The EL peak intensity as a function of the applied voltage exhibited in Figure 11a shows that the heterostructure HTE2 exhibits the lowest turn-on voltage (5 V) and reaches its maximum emission intensity at relatively low bias. However, further increase in the applied voltage (12 V) leads to a rapid decrease in EL intensity, indicating the onset of irreversible degradation. In contrast, the heterostructure HTY4 reaches its highest EL intensity at higher applied voltages (12 V) and maintains emission over a broader voltage range.

The lower turn-on voltage observed for the HTE2 system is consistent with the strong interfacial coordination evidenced by the FTIR spectra shown in Figure 9b, where a bridging bidentate carboxylate configuration was identified. Such organized anchoring is expected to enhance electronic coupling at the organic–inorganic interface, facilitating charge injection at lower applied fields.^55^ The observed device degradation at 12 V, attributed to interfacial effects, can be primarily related to intrinsic processes occurring during device operation, especially in the depletion region close to the interface between both materials. Under higher voltages, there is a greater density of carriers and excitons, which favors undesirable chemical reactions such as bond breaking and the formation of reactive species, such as carboxylate groups coordinated at the interface, which can undergo weakening or bond rupture under an electric field. These processes lead to the emergence of trap states, which act as nonradiative recombination centers, reducing the luminous efficiency.^65^ Additionally, defect states such as oxygen vacancies and intraband gap levels introduced by Er^3+^ doping may act as charge trapping centers, further accelerating degradation through trap-assisted recombination. Therefore, the observed irreversible loss of the EL signal is likely the result of combined interfacial degradation and defect-mediated processes under high bias, explaining the rapid decrease in EL intensity. The internal efficiency of hybrid LEDs also depends on the efficiency of radiative recombination of excitons within the QD active layer and the proper balance of electron fluxes entering from the ETL^66^ and may be induced by the dopants Er^3+^ and Yb^3+^. These findings suggest that rare-earth incorporation alters the interfacial electronic behavior, likely by modulating trap states, charge injection balance, or nonradiative recombination pathways.

In the heterostructure HTY4, although the band corresponding to the carboxylic acid group was not observed, no well-defined carboxylate vibrational modes were detected, suggesting a less organized or distinct coordination environment. This comparatively weaker or more heterogeneous interfacial interaction may reduce the initial electronic coupling, requiring higher applied voltages to achieve maximum EL intensity. On the other hand, such a configuration may relieve interfacial stress under strong electric fields, contributing to the improved operational voltage range observed for this device.

The PL spectra of the heterostructure (inset of Figure 11b) show a blue shift from 500 nm, characteristic of the individual film of QD (Figure 7b), to approximately 483 nm after deposition on rare-earth-doped TiO_2_ film. This shift indicates a modification of the emissive environment upon heterostructure formation.^47^ Possible contributions include reduced molecular aggregation, changes in the local dielectric environment, or interfacial electronic interactions. Notably, no detectable PL was observed for the individually doped TiO_2_ films, confirming that the emission originates from the organic layer and is influenced by its interaction with the oxide surface.

## 4 Conclusion

The accomplished results demonstrate that rare-earth doping of TiO_2_ plays a significant role in controlling the interfacial chemistry and electroluminescence behavior of hybrid TiO_2_/QD heterostructures. Structural analyses confirmed that Er^3+^ and Yb^3+^ incorporation preserves the anatase phase while subtly modifying the crystallographic orientation and surface morphology. Optical measurements revealed defect-related absorption features and characteristic lanthanide transitions, indicating the introduction of localized states with intrabandgap energy within the oxide matrix.

FTIR spectroscopy provided direct evidence of interfacial chemical interaction through the deprotonation of the quinoline carboxylic group and coordination to surface Ti sites. Specifically, Er^3+^-doped TiO_2_ promotes a well-defined bridging bidentate configuration, associated with stronger electronic coupling at the interface. This organized anchoring correlates with the lower turn-on voltage observed in the electroluminescence measurements. On the other hand, Yb^3+^-doped systems exhibit a more heterogeneous coordination environment, requiring higher applied bias but showing improved emission under strong electric fields.

The observed blue-shift in photoluminescence upon heterostructure formation confirms that the light-emitting state of the organic layer is significantly influenced by interfacial electronic interactions. Collectively, the results indicate that lanthanide doping does not merely alter bulk TiO_2_ properties but actively tunes the charge injection balance and recombination processes at the organic–inorganic junction.

The coupling of the quinoline derivative with rare-earth-doped TiO_2_ forms a functional hybrid p–n junction capable of emitting light when photoexcited or electro-excited as well, demonstrating the feasibility of employing this system in the development of low-cost hybrid LEDs processed via chemical routes. Moreover, the obtained results show that lanthanide doping actively remodels surface coordination and modulates interfacial charge injection, establishing the incorporation of rare earths as a strategy for interface-driven electronic control. This control opens opportunities for the design of hybrid emitters in which the optical response not only depends on the material but is also driven by the interface design. The demonstrated coupling mechanism suggests broader applicability in optoelectronic devices where charge transfer states and interfacial dipoles govern performance.

## Supplementary Material



## Notes

^1^H and ^13^C spectrum of quinoline derivative compounds; SEM images of TiO_2_ samples doped with 2% Er^3+^, 2% Yb^3+^, and 4% Yb^3+^; EDS maps for Ti, O of TiO_2_ samples doped with 2% Er^3^ and 2% Yb^3+^; particle size distribution evaluated from SEM images for samples TE2 and TY4; evaluation of texture coefficient for rare-earth doped TiO_2_ thin films; and evaluation of doping concentration in distinct regions for samples TE2, TE4, TY2, and TY4 (PDF)
