# Supplementary material for: Coupling of a Quinoline Derivative and Rare-Earth-Doped TiO2 Thin Films: Prospective Application to Hybrid Light-Emitting Devices
Source: ACS Omega. 2026 Jun 20;11(26):39031–44. doi: 10.1021/acsomega.6c02539 (PMC13347331; doi:10.1021/acsomega.6c02539)
Supplement: Supplementary file 1 [file ao6c02539_si_001.pdf]

Coupling of Quinoline Derivative and Rare-Earth Doped TiO_2 Thin Films: Prospective application to Hybrid Light-Emitting Devices

Natália Carli de Oliveira [1], Xavier Mateos [2], Vitor Fernandes Moreno [3], Luiz Carlos da Silva Filho [3], Luis Vicente de Andrade Scalvi [4*]

[1] São Paulo State University UNESP, School of Sciences. Chemistry Dept. and POSMAT, Av. Luiz Edmundo C. Coube 14-01, 17033-360, Bauru, Brazil

[2] University Rovira i Virgili, Physical and Inorganic Chemistry Dept., Tarragona 43007, Spain

[3] São Paulo State University UNESP, School of Sciences. Chemistry Dept., Av. Luiz Edmundo C. Coube 14-01, 17033-360, Bauru, Brazil

[4*] São Paulo State University UNESP, School of Sciences, Physics and Meteorology Dept., Av. Luiz Edmundo C. Coube 14-01, 17033-360, Bauru, Brazil, scalvi@fc.unesp.br

Supplementary Material

Characterization Data of Quinoline Derivative

4-(6-(diethylamino)-4-phenylquinolin-2-yl)benzoic acid: Light orange solid. **mp/ºC** = 237-238. **^{1}H NMR** (CDCl3, 400 MHz): 8.37 (AA'XX', 2H), 8.06 (AA'XX', 2H), 7.99 (m, 2H), 7.90 (s, 1H), 7.70-7.52 (m, 5H), 6.77 (d, J = 2.8 Hz, 1H), 3.37 (q, J_1 = 6.9 Hz, J_2 = 13.9 Hz, 4H), 1.09 (t, $J_1 = J_2$ = 6.9 Hz, 6H) ppm; **^{13}C NMR** (CDCl3, 100 MHz): δ 149.5, 145.9, 145.5, 141.6, 138.3, 130.9, 129.7, 129.3, 128.8, 128.6, 128.3, 127.2, 126.5, 119.2, 118.9, 100.9, 44.1, 12.4 ppm; **IR** (neat): ν_{max}= 819, 1150, 1265, 1291, 1422, 1513, 1583, 1619, 1680, 2969 cm-1. **ESI–HRMS**: m/z calcd for C26H24N2O2 $[M + H]^+$: 397.1911; found 397.1919.

This report was created by ACD/NMR Processor Academic Edition. For more information go to www.acdlabs.com/nmrproc/

Acquisition Time (sec)	3.9584						
Frequency (MHz)	400.13	***Nucleus***	1H	***Number of Transients***	16	***Origin***	spect
Original Points Count	32768	***Owner***	nmrsu	***Points Count***	32768	***Pulse Sequence***	zg30
Receiver Gain	11585.20	***SW(cyclical) (Hz)***	8278.15	***Solvent***	DMSO-d6	***Spectrum Offset (Hz)***	2467.8215
Spectrum Type	STANDARD	***Sweep Width (Hz)***	8277.89	***Temperature (degree C)***	27.000		

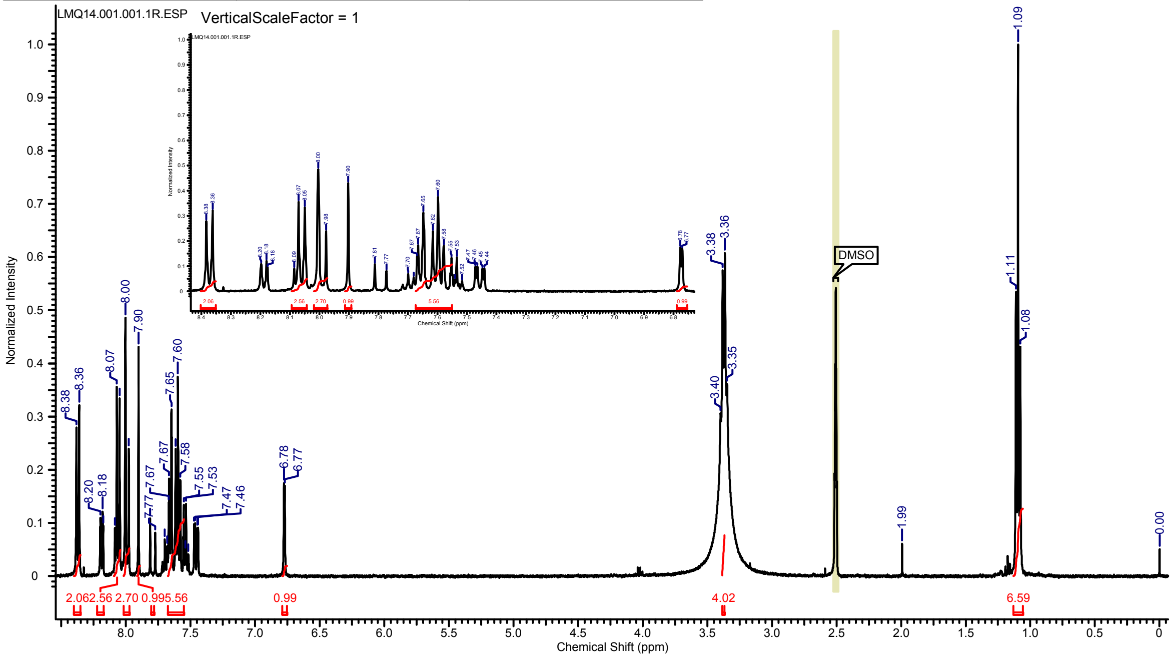

This report was created by ACD/NMR Processor Academic Edition. For more information go to www.acdlabs.com/nmrproc/

Acquisition Time (sec)	1.3664						
						Frequency (MHz)	100.61
Nucleus	13C	***Number of Transients***	2560	***Origin***	spect	***Original Points Count***	32768
Owner	nmrsu	***Points Count***	32768	***Pulse Sequence***	zgpg30	***Receiver Gain***	1625.50
SW(cyclical) (Hz)	23980.81	***Solvent***	DMSO-d6	***Spectrum Offset (Hz)***	10010.4971	***Spectrum Type***	STANDARD
Sweep Width (Hz)	23980.08	***Temperature (degree C)***	27.000				

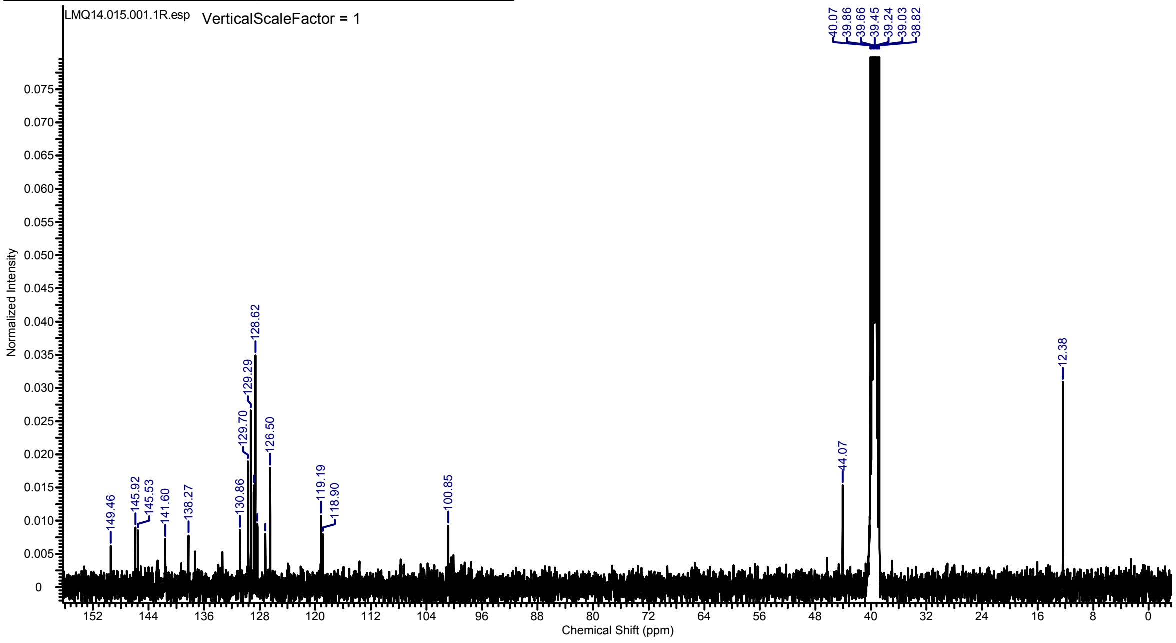

Figure S1. ^{1}H and ^{13}C spectrum of compounds

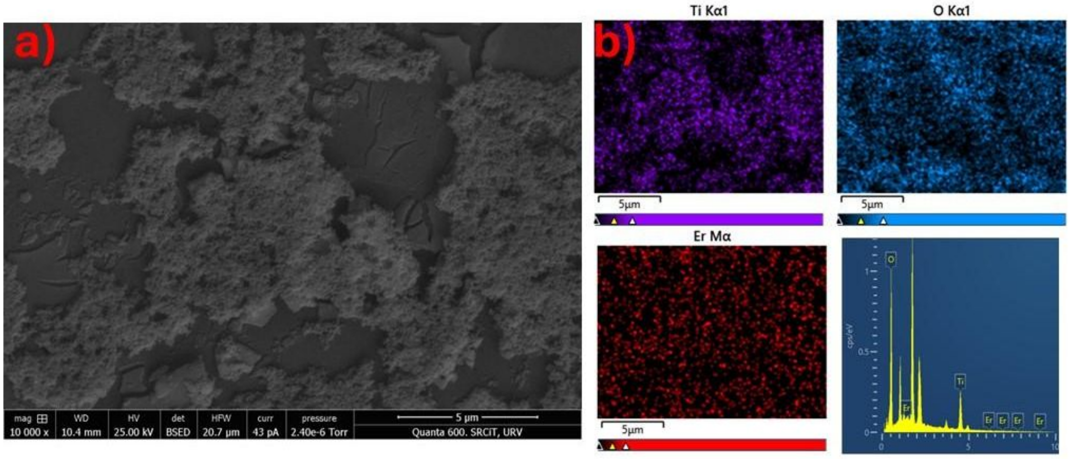

Figure S2. 2% Er^{3+}: TiO_2 a) SEM images. Magnification: 10,000x b) EDS maps for Ti , O and Er

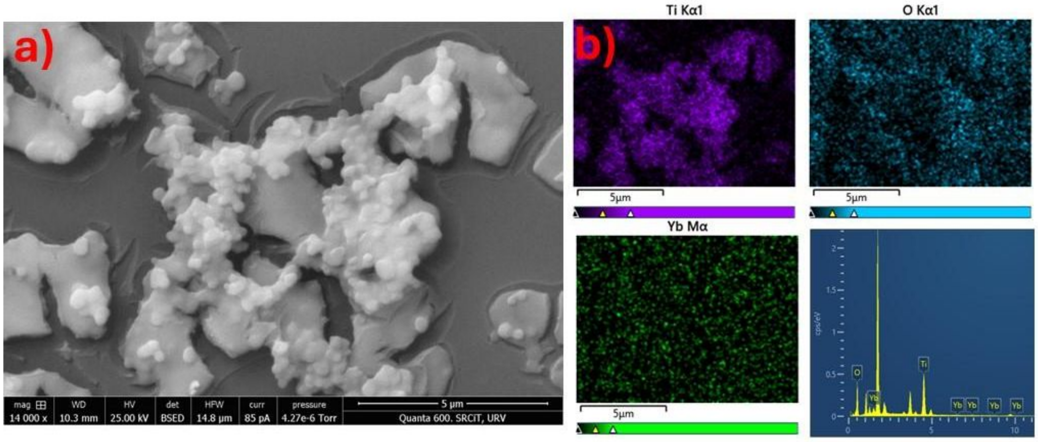

Figure S3. 2% Yb^{3+}: TiO_2 a) SEM images. Magnification: 14,000x **b)** EDS maps for Ti, O and Yb.

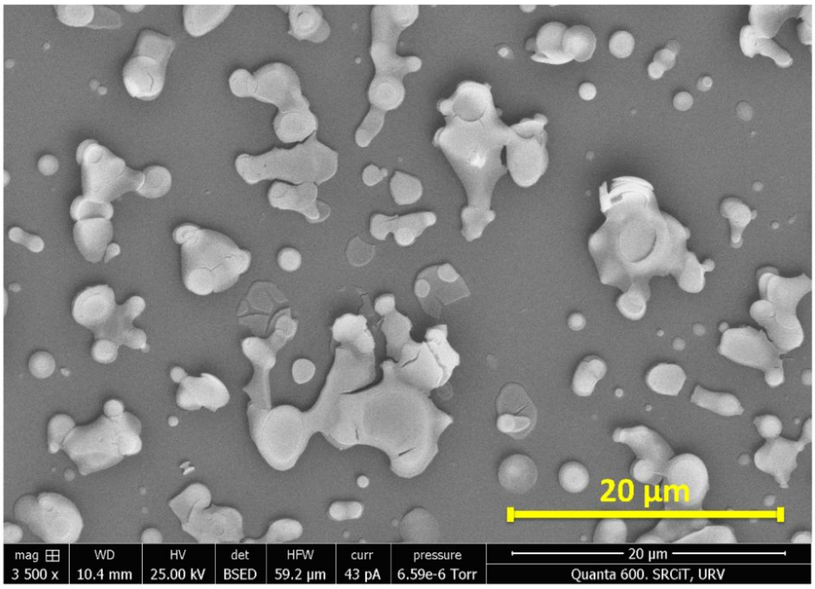

Figure S4. SEM images of 4% Yb^{3+}: TiO_2, magnification: 3,500x

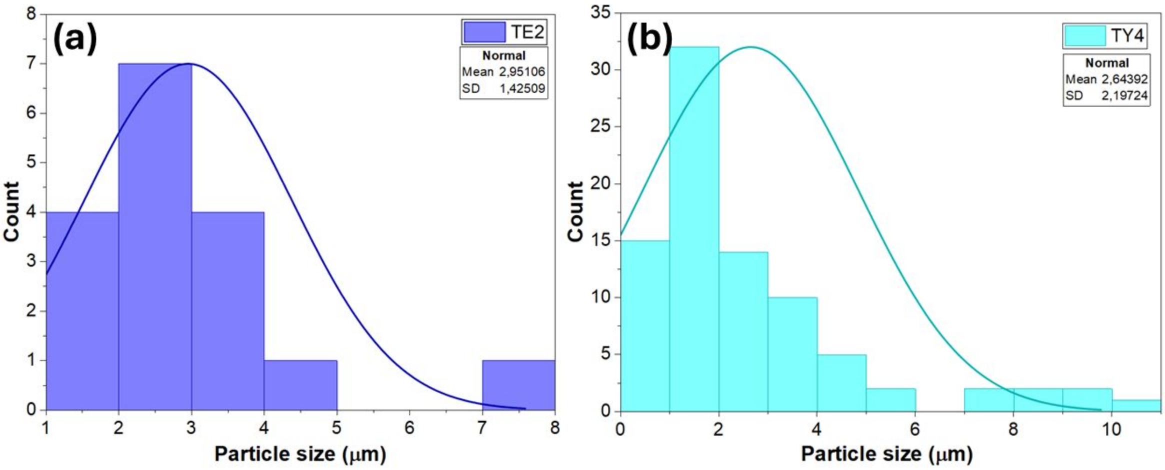

Figure S5. Particle size distribution evaluated from SEM images. **(a)** sample TE2. **(b)** sample TY4

Texture Coefficient

The equation S1 [1] was used to calculate the texture coefficients (CT) of the TiO_2 samples. Considering that the plane (004) appears to be dominant in the XRD diffractograms, the CT was calculated in this direction (perpendicular to the (004) plane) for all the samples. The table S1 shows the results of this calculation.

$$TC(hkl) = \frac{I(hkl)/I_0(hkl)}{\frac{1}{N}\sum_{n=1}^{N} I(hkl)_n/I_0(hkl)_n} \quad \text{(S1)}$$

Where $I(hkl)$ is the intensity of the diffraction peak (hkl) in the sample under investigation; $I_0(hkl)$ is the intensity of the plane (hkl) taken from a diffraction pattern; and N is the number of planes considered in the analysis. A CT much larger than the unity indicates a preferential growth in that direction [1].

As an example of calculation, it is shown below the evaluation carried out for the 2% Yb^{3+}:TiO_2 film (equation S2)

$$TC(004) = \frac{2840.94/1391.91}{\frac{1}{2}\times(2840.94/1391.91 + 2005.51/9958.22)} = 1.82 \quad \text{(S2)}$$

Table S1 – Texture coefficient for rare-earth doped TiO_2 thin films

**TiO_2 Sample**	**CT**	**TiO_2 Sample**	**CT**
Undoped	0.91	0.5% Yb^{3+}	1.78
0.5% Er^{3+}	1.75	1% Yb^{3+}	1.78
1% Er^{3+}:	1,83	2% Yb^{3+}	1.82
2% Er^{3+}	1,82	3% Yb^{3+}	1.77
3% Er^{3+}	1.84	4% Yb^{3+}	1.82
4% Er^{3+}	1.87		

It can be noted that texture appears in relation to plane (004) in all samples with doping added, which seems to follow a tendency of higher CT with higher doping. This indicates that the substitution doping of lanthanide ions by Ti atoms causes significant distortion in the crystal lattice.

Evaluation of doping concentration in distinct regions

The atomic percentage values of lanthanide ions found for the different regions of samples TE2, TE4, TY2, and TY4 are shown in Table S2, as obtained from Scan EDS data.

Table S2. Atomic percentage concentration of lanthanide dopant ions in different regions of samples TE2, TE4, TY2, and TY4.

Region/ Sample	TE2 (Er^{3+} at%)	TE4 (Er^{3+} at%)	TY2 (Yb^{3+} at%)	TY4 (Yb^{3+} at%)
R1	0.79	0.21	1.09	1.60
R2	0.89	0.21	7.60	7.23
R3	-	-	1.41	6.33

Reference

[1] Wang, Y.; Tang, W.; Zhang, L. Crystalline Size Effects on Texture Coefficient, Electrical and Optical Properties of Sputter-deposited Ga-doped ZnO Thin Films, Journal of Materials Science & Technology **2015,** 31(2), 175-181. https://doi.org/10.1016/j.jmst.2014.11.009.
